# Solvolysis of the Tumor-Inhibiting Ru(III)-Complex *trans*-Tetrachlorobis(Indazole)Ruthenate(III)

**DOI:** 10.1155/MBD.2000.225

**Published:** 2000

**Authors:** Thomas Pieper, Wolfgang Peti, Bernhard K. Keppler

**Affiliations:** Institute of Inorganic Chemistry University of Vienna Waehringerstr. 42 Vienna A-1090 Austria

## Abstract

The ruthenium(III) complex Hlnd *trans*-[RuCl_4_,(ind)_2_], with two *trans*-standing indazole (ind) ligands bound to ruthenium via nitrogen, shows remarkable activity in different tumor models *in vitro* and *in vivo*. The solvolysis of the complex *trans*-[RuCl_4_,(ind)_2_]^-^ has been investigated by means of spectroscopic techniques (UV/vis, NMR)in different solvents. We investigated the indazolium as well as the sodium salt, the latter showing improved solubility in water. In aqueous acetonitrile and ethanol the solvolysis results in one main solvento complex. The hydrolysis of the complex is more complicated and depends on the pH of the solution as well as on the buffer system.


### SOLVOLYSIS OF THE TUMOR-INHIBITING Ru(III)-COMPLEX *trans*-TETRACHLOROBIS(INDAZOLE)RUTHENATE(III)

Thomas Pieper, Wolfgang Peti and Bernhard K. Keppler*

Institute of Inorganic Chemistry, University of Vienna, Waehringerstr. 42, A-1090 Vienna, Austria

Abstract
The ruthenium(III) complex HInd *trans*-[$RuCl_4(ind)_2$], with two *trans*-standing indazole (ind) ligands bound to ruthenium via nitrogen, shows remarkable activity in different tumor models *in vitro* and *in vivo*. The solvolysis of the complex *trans*-[$RuCl_4(ind)_2]^-$ has been investigated by means of spectroscopic techniques (UV/vis, NMR) in different solvents. We investigated the indazolium as well as the sodium salt, the latter showing improved solubility in water. In aqueous acetonitrile and ethanol the solvolysis results in one main solvento complex. The hydrolysis of the complex is more complicated and depends on the pH of the solution as well as on the buffer system.

### 1. Introduction

Today, *cisplatin* is a well established chemotherapeutic drug, especially against testicular carcinomas, but expansion to a different or broader antitumor spectrum has not been obtained with cisplatin or direct analogues, like carboplatin. Some new developments like orally administrable platinum complexes or combination therapy are promising, but attention is more and more directed to non-platinum antitumor metal compounds. Among others, ruthenium complexes of the oxidation state +II and +III are under current investigation as alternative drugs to platinum-based tumor inhibitors [1,2,3,4]. Because of their different chemical characteristics and kinetics, the mode of action and spectrum of activity of these ruthenium compounds should differ significantly from the known platinum complexes. They possess a different redox behavior and undergo different hydrolysis reactions. As a result, a different interaction with biological targets can be expected and detected.
Especially ruthenium(III) complexes of the general formula HL[$RuCl_4L_2$], with two *trans*-standing heterocyclic ligands L bound to ruthenium via nitrogen, show remarkable activity in different tumor models *in vitro* and *in vivo*. They exhibit excellent activity in an autochthonous colorectal tumor model, which is comparable to human colon tumors in its histological appearance and behavior against chemotherapeutics, with a tumor reduction of about 70% to 90% [5,6]. *Cisplatin* is completely inactive in this model. Furthermore, these Ru(III)-complexes show antiproliferative activity in two human colon cancer cell lines (SW707 and SW948) [7]. The most promising complex contains two *trans*-standing indazole (L = ind) ligands. It exhibits antineoplastic effects on proliferation of clonogenic cells from freshly explanted human tumors in a capillary soft agar cloning system [8]. Compared to the also very active imidazole (L = im) complex it is less toxic. The slightly different activity and significantly different toxicological profile of the imidazole and indazole complexes might be due to their different hydrolysis reactions and kinetics and binding preferences for biological targets. Aquation is an important step in the activation of cisplatin [9] and aqua complexes, in general, are orders of magnitude more labile than the corresponding chloro complexes [10,11,12]. It is also known that only aged aqueous solutions of HIm *trans*-[$RuCl_4(im)_2$] react with DNA [13]. Therefore, the knowledge of hydrolysis or, in general, solvolysis products is of great importance for a deeper understanding of the mode of action of the ruthenium complexes.

The hydrolysis reactions of the imidazole complex HIm *trans*-[$RuCl_4im_2$] have already been investigated by means of $^1$H-NMR, EPR or UV/vis spectroscopy [14,15,16]. In each case hydrolysis to a monoaqua complex has been proposed in unbuffered aqueous solution, followed by the formation of both of the possible diaqua complex isomers with *trans*-standing imidazoles. The situation in phosphate-buffered solution was found to be more complicated.
Here we report on the solvolysis in ethanol, acetonitrile and dimethylsulfoxide and the hydrolysis of the indazole complex *trans*-[$RuCl_4(ind)_2]^-$ (see figure 1a). We investigated the indazolium as well as the sodium salt, the latter showing improved solubility in water.

### 2. Materials and Methods

Solvents used were of analytical grade and dried with standard methods.
Elemental analysis C,H,N analysis were carried out on a Perkin Elmer 2400 CHN Elemental Analyzer. Cl was analyzed by argentometry.
Spectrophotometric experiments were performed in a thermostated cell compartment in a Perkin Elmer Lambda 20 UV/vis spectrometer.
$^1$H NMR experiments were performed at 400.13 MHz ($^1$H) on a Bruker DPX 400 spectrometer.
HInd *trans*-[$RuCl_4(ind)_2$] [17] and Na *trans*-[$RuCl_4(ind)_2$]·$3H_2O$ [18] were prepared as described elsewhere. To obtain "water-free" Na *trans*-[$RuCl_4(ind)_2$] for reactions in absolute solvents, the trihydrate is dissolved in acetonitrile and the complex salt precipitated by addition of diethylether and dried under vacuum. $C_{14}H_{12}Cl_4N_4NaRu$ (502.15): calcd. C 33.49, H 2.41, N 11.16; found: C 33.33, H 2.66, N 11.05. $^1$H-NMR (400 MHz, [$D_3$] acetonitrile): δ 4.31 (2 H), 3.18 (2 H), 2.63 (2 H), 2.44 (2 H), -7.1 (2 H), -12.9 (2 H) ppm. $^{13}$C-NMR (100 MHz, acetonitrile-d3): δ 118.05, 114.71, 98.27, 97.75 ppm.
Precipitate obtained from a solution of Na *trans*-[$RuCl_4(ind)_2$]·$3H_2O$ in water: The dark solid that precipitated in a nearly saturated solution of Na *trans*-[$RuCl_4(ind)_2$]·$3H_2O$ in water at room temperature was filtered after one week. Elemental analysis, found: C 36.47, H 2.96, N 11.85, Cl 21.95, calculated for [$RuCl_3(H_2O)(ind)_2$], $C_{14}H_{14}Cl_3N_4ORu$ (461.72): C: 36.42, H 3.06, N 12.13, Cl 23.04. $^1$H-NMR (400 MHz, dmso-d6): δ 4.49, 4.19, 3.71, 3.64, -4, -10 ppm and, with 2% relative peak area, 7.17, 7.40, 7.80, 8.75 ppm.
Precipitate obtained from a solution of Na *trans*-[$RuCl_4(ind)_2$]·$3H_2O$ in acetonitrile / water: The dark solid that precipitated in a nearly saturated solution of Na *trans*-[$RuCl_4(ind)_2$]·$3H_2O$ in acetonitrile / water (70/30) at room temperature was filtered after one week. Elemental analysis, found: C: 39.71, H 2.95, N 14.37, Cl 21.86, calculated for [$RuCl_3(CH_3CN)(ind)_2$], $C_{16}H_{15}Cl_3N_5Ru$ (484.76): C 39.64, H 3.12, N 14.45, Cl 21.94. $^1$H-NMR (400 MHz, $CDCl_3$): δ 9.09, 5.59, 5.10, 4.40, 4.04, 2.89, 1.09, -2.22, -12 and –17 ppm.

### 3. Results and Discussion

### *3.1. Solvolysis of Na trans-[$RuCl_4(ind)_2$]*

Na *trans*-[$RuCl_4(ind)_2$] does not react in acetonitrile within two days. No changes in $^1$H NMR- and UV/vis-spectra can be seen. In the $^1$H NMR spectrum of Na *trans*-[$RuCl_4(ind)_2$] the typical line broadening and chemical shifts due to the paramagnetic Ru(III)-center can be observed [19,20]. The most upfield shifted signal at a chemical shift of -13.0 ppm represents the two protons at position 3 of the two equivalent indazole ligands. The signal at -7.1 ppm can be assigned to each proton at N-2 because it disappears, due to rapid exchange, on addition of $D_2O$. The less broad and upfield shifted peaks at 2.45 (2 h at C-4 or C-7), 2.63 (2 H at C-7 or C-4), 3.18 (2 H at C-5 or C-6) and 4.32 ppm (2 H at C-6 or C-5), that can be assigned in pairs because of corresponding cross-peaks in the H,H-COSY experiment, represent the protons of the indazole ligands with a greater distance to the paramagnetic Ru(III)-center [18].
The possibility that the spectra in a fresh solution of Na *trans*-[$RuCl_4(ind)_2$] do not represent the structure determined for the solid complex salt [18], could be ruled out by evaporating the solvent or precipitating the complex salt with diethylether and characterizing the obtained product again. No changes in the IR- or $^1$H NMR-spectra could be observed.
If a mixture of acetonitrile / water is used instead of pure acetonitrile, a transformation can be seen in $^1$H NMR- and UV/vis-spectra. Na *trans*-[$RuCl_4ind_2$] reacts in acetonitrile / water (70/30), the eluent mixture that is used as solvent in HPLC experiments of the compound, to a main solvolysis product. Figure 1b shows the changes in $^1$H NMR-spectra taken during 72 hours at room temperature. Four new peaks at a chemical shift of 3.77, 3.56, 0.44 and 0.11 ppm show up while the intensity of the peaks representing the „original" complex *trans*-[$RuCl_4(ind)_2$]$^-$ at 3.65 (overlapping the new peak at 3.77), 3.10, 2.34 and 2.18 ppm decreases. The upfield shifted peak at -14.9 disappears. No other signal could be observed. Precipitation occurs after one week at room temperature. This precipitation was only observed at the concentrations used in the NMR experiments. The precipitate is soluble in less polar solvents like chloroform. Although the elemental analysis of the precipitate fits the calculated values for the neutral monosolvento complex [$RuCl_3(CH_3CN)(ind)_2$], the $^1$H NMR data are not consistent with this proposal. At least eight signals of nearly equal intensity between 9 and -2 ppm and two broad signals at -12 and -17 ppm could be detected. Information from COSY spectra are limited due to the paramagnetic Ru(III) center but coupling between four of the ten signals could be found.

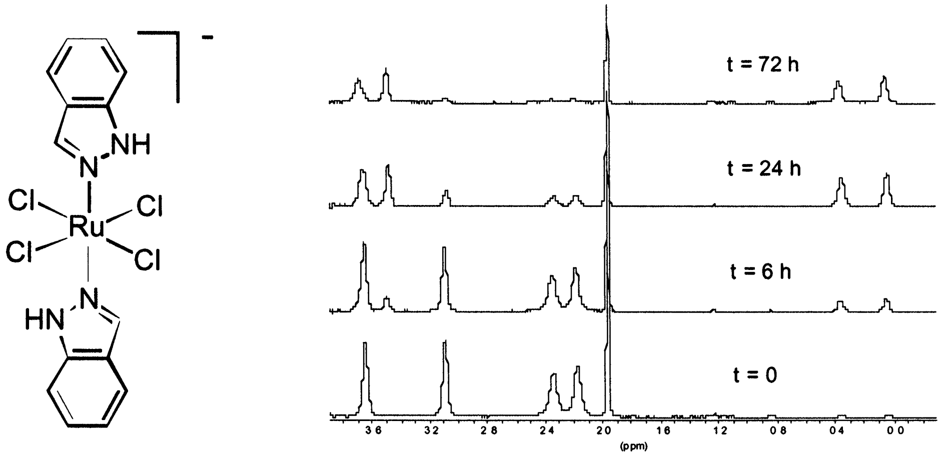

a b

**Figure 1.** a) Tumor-inhibiting Ru(III) complex *trans*-$[RuCl_4ind_2]^-$, b) $^1H$ NMR-spectra of Na *trans*-$[RuCl_4(ind)_2]$ in $CD_3CN$ / $D_2O$ (70/30) during 3 days at room temperature between a chemical shift of 0 to 3.8 ppm.

The changes observed in the UV/vis-spectra also confirm the formation of one main solvolysis product of Na *trans*-$[RuCl_4ind_2]^-$ in acetonitrile / water mixtures. The original spectrum with maxima at 239, 292 and 373 nm changes to give a spectrum with maxima at 279, 379, 398, 536 and 635 nm after 24 hours at 37°C, as can be seen in figure 2a. Isosbestic points at 217, 282 and 314 nm indicate a definite transformation into one main solvolysis product. The changes in the UV/vis-spectra are significantly different from those in pure water (see below). Thus, solvolysis of Na *trans*-$[RuCl_4(ind)_2]$ does not occur in absolute acetonitrile solution within days, whereas it takes place in acetonitrile / water mixtures. One explanation for these findings could be an initial, rate-determining reaction of *trans*-$[RuCl_4(ind)_2]^-$ with water, presumably leading to the monoaquacomplex $[RuCl_3(H_2O)(ind)_2]$. The monoaquacomplex could then react with acetonitrile, forming an acetonitrile complex $[RuCl_3(CH_3CN)(ind)_2]$, as could be demonstrated in similar cases [21].

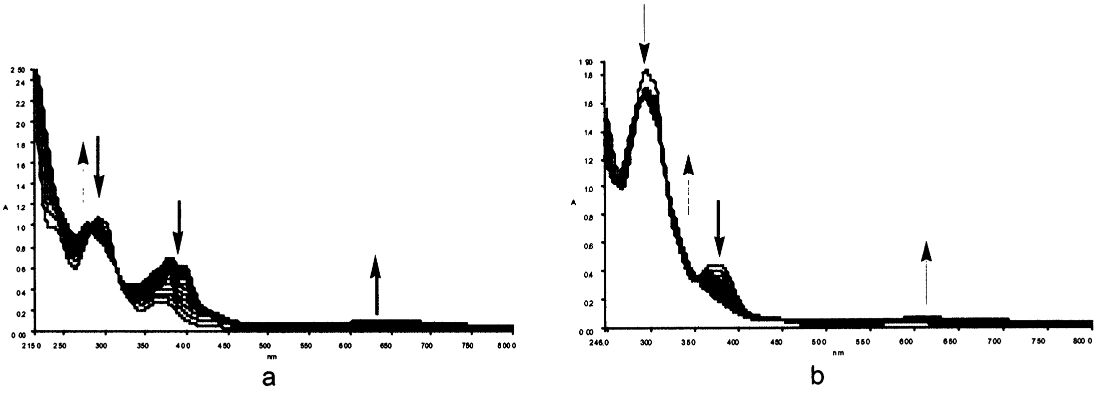

a b

**Figure 2.** a) UV/vis-spectra (215-800 nm) of Na *trans*-$[RuCl_4(ind)_2]$ in acetonitrile / water (70/30) during 48 hours at room temperature; b) UV/vis-spectra (250-750 nm) of Na *trans*-$[RuCl_4(ind)_2]$ in dmso / water (1/1) during 12 hours at 37°C.

As in the case of acetonitrile, no change in the $^1H$ NMR of Na *trans*-$[RuCl_4(ind)_2]$ can be observed in absolute dmso-d6 solution within a week. The signals at -11.0 (2 H at C-3), -6.1 (2 H at N-2), 2.49 (2 H at C-4 or C-7), 3.22 (4 H at C-7 or C-4 and C-5 or C-6) and 4.40 ppm ( 2 H

at C-6 or C-5) remain unchanged in intensity and chemical shift. The UV/vis-spectra in absolute dmso don't give any evidence for a transformation of the ruthenium complex either.
In contrast, the spectra of Na *trans*-$[RuCl_4(ind)_2]$ change significantly in dmso / water mixtures. The UV/vis spectrum after 12 hours at 37°C is totally different from the spectrum in acetonitrile / water and also different from the spectrum in water (see below). After one hour a band occurs at 610 nm (in water at 580 nm), whereas the band at 377 nm disappears, as can be seen in figure 2b. Isosbestic points can be found at 261, 283, 312 and 354 nm. No precipitation occurs.
The $^1$H NMR experiment in dmso-d6 / $D_2O$ does not lead to further information. No changes in chemical shifts, only a decrease in intensity of the signals due to precipitation of a dark solid can be observed.
The reaction of Na *trans*-$[RuCl_4(ind)_2]$ in ethanol can be monitored by UV/vis- and NMR-spectroscopy. Figure 3a shows the changes in the $^1$H NMR spectra of a solution of Na *trans*-$[RuCl_4(ind)_2]$ in ethanol-d4 in the chemical shift region of 2.0 to 4.2 ppm. The spectrum at the bottom represents the $^1$H NMR of Na *trans*-$[RuCl_4(ind)_2]$ just after dissolving in deuterated ethanol. The peaks of the original complex anion appear at a chemical shift of 2.24, 2.49, 3.19 and 3.54 ppm. After two hours at 37°C a peak at 3.13 ppm and two signals at 3.88 and 3.98 ppm arise (they can already be seen in the first spectrum), whereas the intensity of the original peaks decreases. The increase in intensity of the new downfield-shifted signals reaches a plateau after about 12 hours. The signal intensity ratio, original complex / solvolysis product, stays at about 1/3 even after one week. The signal pattern can be attributed to one solvolysis product, as the increase in intensity is simultaneous and coupling of the protons of this solvolysis product can be determined with a H,H-COSY experiment (data not shown). The peaks at 3.13 and 3.98 ppm represent one proton each, the peak at 3.88 ppm represents two protons of each indazole ligand. Changes in $^1$H NMR spectra also occur simultaneously in the down field region. The broad signal of the proton at position 3 of the indazole ligand of the original complex anion *trans*-$[RuCl_4(ind)_2]^-$ at -14.3 ppm disappears, whereas a new signal rises at -11.3 ppm (see figure 3b). In the aromatic region multiplets at 7.26, 7.51, 7.74, 7.84 ppm and a singlet at 8.86 can be detected after six hours (data not shown), showing the typical signal pattern for indazole. The signals are of very low intensity (only about 2% of the main solvolysis product even after a week) and can be attributed to indazole ligands coordinated to diamagnetic ruthenium centers as they are significantly downfield shifted compared to those of free indazole in ethanol-d4 (7.10, 7.34, 7.54, 7.73, 8.00 ppm).

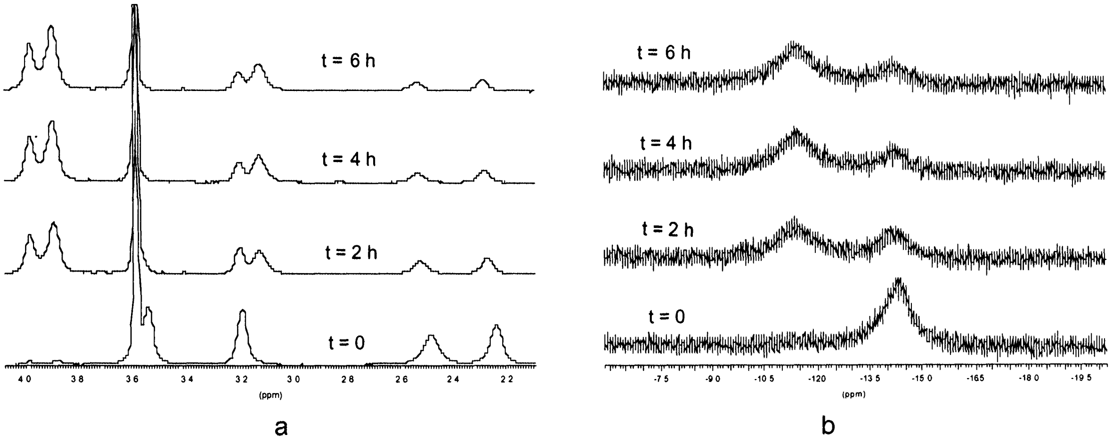

**Figure 3.** $^1$H NMR-spectra of Na *trans*-$[RuCl_4(ind)_2]$ in ethanol-d6 after 0, 2, 4 and 6 hours at 37°C between a chemical shift of a) 2.1 and 4.0 ppm (left) and b) -7 and -20 ppm (right).

The transformation into one main solvolysis product is also obvious in the UV/vis spectra taken at a temperature of 37°C during the first six hours (see figure 5a). The transformation of the original complex is characterized by isosbestic points at 261, 304, 339, 355 and 404 nm. No band above 415 nm can be detected. No further changes, especially no precipitation can be observed within two days of observation period.

In an ethanol / water mixture (1/1) the changes in the UV/vis-spectra are different. In contrast to the spectra in pure ethanol, a band rises at 578 nm (see figure 5b). The transformation of the complex is characterized by isosbestic points at 276, 310, 361 and 445 nm. Whereas hydrolysis of Na *trans*-[$RuCl_4(ind)_2$] in pure water leads to precipitation (see below), this can not be observed in ethanol / water mixtures.

Four signals at a chemical shift of -17.9, 1.27, 2.09, 2.41 and 2.80 ppm can be detected in the $^1H$ NMR spectrum for the complex *trans*-[$RuCl_4(ind)_2]^-$, just after dissolving the sodium salt in ethanol / water (1/1). The signals are slightly shifted compared to pure $D_2O$ (see below). New signals arise at 1.68, 1.87 and 3.18 ppm but their intensity never exceeds one third of the signals of the „fresh" solution. After three hours a precipitation starts that is finished after eight hours.

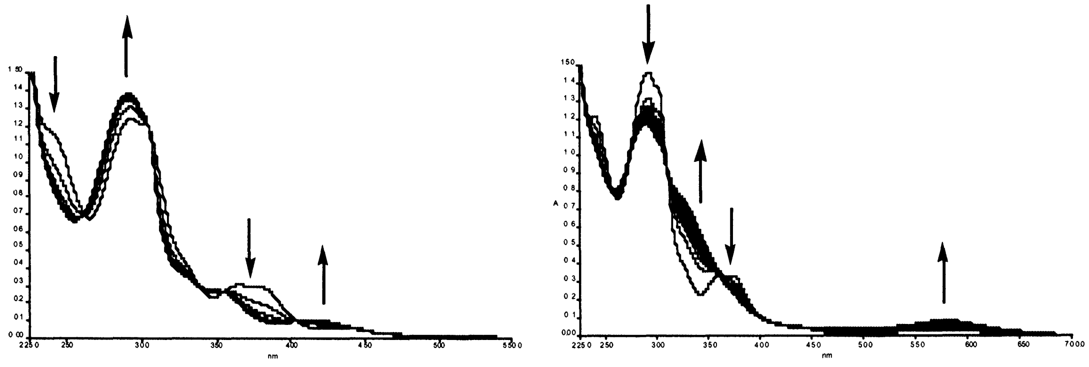

a b

**Figure 4.** UV/vis-spectra of Na *trans*-[$RuCl_4(ind)_2$] in a) ethanol during 6 hours at 37°C (225-550 nm) and b) ethanol / water (1/1) during 12 hours at 37°C (225-700 nm).

HInd *trans*-[$RuCl_4(ind)_2$] reacts quite similar to Na *trans*-[$RuCl_4(ind)_2$]. We found no evidence for the interference of the counterion or the formation of the neutral trisindazole complex *mer*-[$RuCl_3(ind)_3$] under the conditions mentioned. *mer*-[$RuCl_3(ind)_3$] can only be obtained on refluxing a suspension of HInd *trans*-[$RuCl_4(ind)_2$] in solvents like tetrahydrofuran or ethyl acetate (to be published).

### *3.2. Hydrolysis of Na trans-[RuCl₄(ind)₂]*

The hydrolytic reactions of the imidazole complex HIm *trans*-[$RuCl_4(im)_2$] have already been investigated by means of $^1H$ NMR spectroscopy, although the information derived from NMR techniques is limited due to the paramagnetic Ru(III) center. In unbuffered solutions of HIm *trans*-[$RuCl_4(im)_2$] new signals that have been assigned to a monoaqua complex and the two possible diaqua complexes arised. Interpretation of the more complicated spectra obtained in phosphate-buffered solutions was not possible [16].

In contrast to the imidazole complex, no changes in chemical shifts can be observed if a solution of the indazole complex Na *trans*-[$RuCl_4(ind)_2$] in $D_2O$ is investigated with $^1H$ NMR spectroscopy (The solubility of the indazolium salt HInd *trans*-[$RuCl_4(ind)_2$] is not good enough for $^1H$ NMR studies). However, precipitation occurs soon and the intensity of the unchanged, paramagnetically shifted peaks at -18.3, 1.30, 2.18, 2.47 and 3.04 ppm decreases. Thus, at least one hydrolysis product is formed which immediately precipitates and therefore can't be detected in solution with NMR. A $^1H$ NMR of the precipitate dissolved in dmso-d6 shows signals of the same intensity at a chemical shift of 3.64, 3.71, 4.19 and 4.49 ppm presumably belonging to one single paramagnetic species. Additional sharp peaks of much lower intensity (under 2%) occur in the aromatic region at 7.17, 7.40, 7.80 and 8.75 ppm. These signals should derive from coordinated indazole protons of diamagnetic species. In the upfield region two very broad signals at -4 and -10 ppm can be detected.

One possible hydrolysis product would be the monoaqua complex [$RuCl_3(H_2O)(ind)_2$] or the corresponding hydroxo complex, respectively. The neutral monoaqua complex should be less soluble in water. If it is withdrawn from solution by precipitation, no further hydrolysis reaction could take place and be detected in solution. Nevertheless, the formation of soluble hydrolysis products without NMR-detectable protons, like those of the indazole ligands, can not be ruled out (see UV/vis experiments). An elemental analysis of the precipitate of Na *trans*-$[RuCl_4(ind)_2]$ in water is within a tolerable margin for C, H and N but the Cl content differs by 1.1% from the value calculated for the monoaquacomplex. The crystal structure of the monoaqua complex of the N-1 methylated indazole complex $[RuCl_3(H_2O)(1\text{-Me-ind})_2]$ could already be solved [17]. The crystals could be obtained by evaporating a solution of H(1-Me-ind) *trans*-$[RuCl_3(H_2O)(1\text{-Me-ind})_2]$ in acetone / water (1/1).

In contrast to the NMR experiments, changes of a solution of Na *trans*-$[RuCl_4(ind)_2]$ in water can be seen in the UV/vis-spectra. Figure 5a shows the changes in the UV/vis spectra during the first eight hours at 37°C. Together with the formation of a band at 578 nm a diffused background absorption caused by precipitation can be observed, resulting in an increased baseline. Nevertheless, one has to be aware of the more than 100 times smaller concentration of Na *trans*-$[RuCl_4(ind)_2]$ in the solution of the UV/vis compared to the NMR experiment. Therefore, different hydrolysis pathways can not be excluded.

Hydrolysis, of course, proceeds slower at room temperature or at 4°C, where no changes in UV/vis spectra can be observed even after days. This is important to notice with regard to a clinical application and storage of solutions of Na *trans*-$[RuCl_4(ind)_2]$.

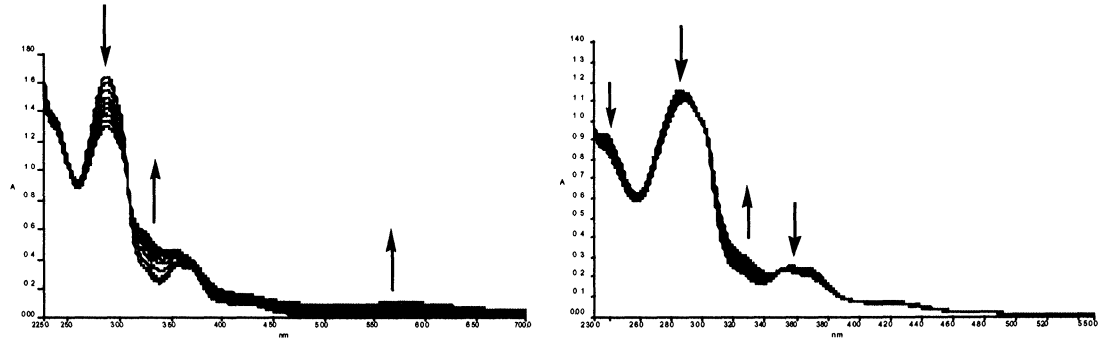

a b

**Figure 5.** a) UV/vis spectra (225-670 nm) of Na *trans*-$[RuCl_4(ind)_2]$ in aqueous solution at 37°C during the first 8 hours; b) UV/vis spectra (225-550 nm) of Na *trans*-$[RuCl_4(ind)_2]$ at 37°C over 10 min in a buffer consisting of 0.004 M $NaH_2PO_4$, 0.025 M $NaHCO_3$ and 0.1 M NaCl.

Reaction of Na *trans*-$[RuCl_4(ind)_2]$ in a solution buffered to pH 7.4 with phosphate buffer is faster than in pure water ($D_2O$). $^1H$ NMR spectra give only little information about the hydrolytic process as precipitation occurs even faster and only a decrease in intensity but no change in the chemical shifts of the protons assigned to the complex *trans*-$[RuCl_4(ind)_2]^-$ can be detected. The dark precipitate is poorly soluble in dmso. The $^1H$ NMR in dmso-d6 reveals a number of signals similar to those of the precipitate obtained in pure water and additional four small signals between 11.5 and 13 ppm. The signals between 7.1 and 8.8 ppm are more intense as in the case of the pure water solution (about 25%). The UV/vis spectra are quite similar to those for pure water, revealing the formation of a band at 578 nm and precipitation. Hydrolysis of the complex *trans*-$[RuCl_4(ind)_2]^-$ is much slower at lower pH levels, whereas it is significantly faster at higher pH levels. If the pH of a solution of *trans*-$[RuCl_4(ind)_2]^-$ at 37°C is adjusted to 3.5 with orthophosphoric acid, the transformation of the complex is indicated by intensity changes of the bands at 287, 357 and 417 nm and isosbestic points at 272, 308 and 344 nm in the UV/vis spectra during the first five hours after dissolving the complex. After this initial transformation further changes in the UV/vis spectra can be detected, characterized by isosbestic points at 357, 464 and 647 nm and the formation of a new band at 578 nm. The band at 578 nm reaches its maximum after 60 h at 37°C, followed by a decrease in absorbance in the whole wave length region because of precipitation. This band at 578 nm is a good indicator to demonstrate the pH-dependence of the hydrolysis. Figure 6a shows a plot of the time after first appearence of the band at 578 nm in the visible spectra against the pH of buffered solutions of Na *trans*-$[RuCl_4(ind)_2]$.

Na *trans*-$[RuCl_4(ind)_2]$ and HInd *trans*-$[RuCl_4(ind)_2]$ show a total different behaviour if $NaHCO_3$ is present in aqueous solution or if a physiological buffer, consisting of 0.004 M $NaH_2PO_4$, 0.025 M $NaHCO_3$ and 0.1 M NaCl, is used, respectively. A solution of Na *trans*-$[RuCl_4(ind)_2]$ in this

$HCO_3^-$ containing buffer (pH 7.4) changes color to green and further on to green-blue within minutes and precipitation occurs after 15 minutes. Figure 6b shows repetitive scan spectra during the first ten minutes, revealing the formation of one new species, characterized by isosbestic points at 298 and 350 nm. After standing for one day the solution is totally colorless. The $^1H$ NMR of the precipitate dissolved in dmso-d6 reveals sharp signals at 8.06 [s, H at C-3], 7.74[m, H at C-4], 7.52 [m, H at C-7], 7.32 [m, H at C-5], 7.08 [m, H at C-6] and 13.06 ppm that fit perfectly the chemical shifts of the indazolium ion in dmso-d6 solution. These signals are overlapped by a very broad signal centered at 7.2 ppm (see figure 6b). No other signals could be detected. Thus, the indazole ligands seem to be released, at least in part. Whether the precipitate consists of a monomeric, dimeric or polymeric species or a mixture of species can not be ruled out based on these investigations.

Important to notice is the fact that $HCO_3^-$ has no comparable influence on the hydrolysis of HIm *trans*-$[RuCl_4(im)_2]$. Whereas hydrolysis of the imidazole complex is faster in water or phosphate buffer, the situation changes dramatically in buffer systems containing hydrogen carbonate. In these buffer systems also binding towards serum proteins was found to be faster.

If a citrate / phosphate buffer is used a similar behaviour can be observed, probably due to reaction of the ruthenium center with the carboxylato groups.

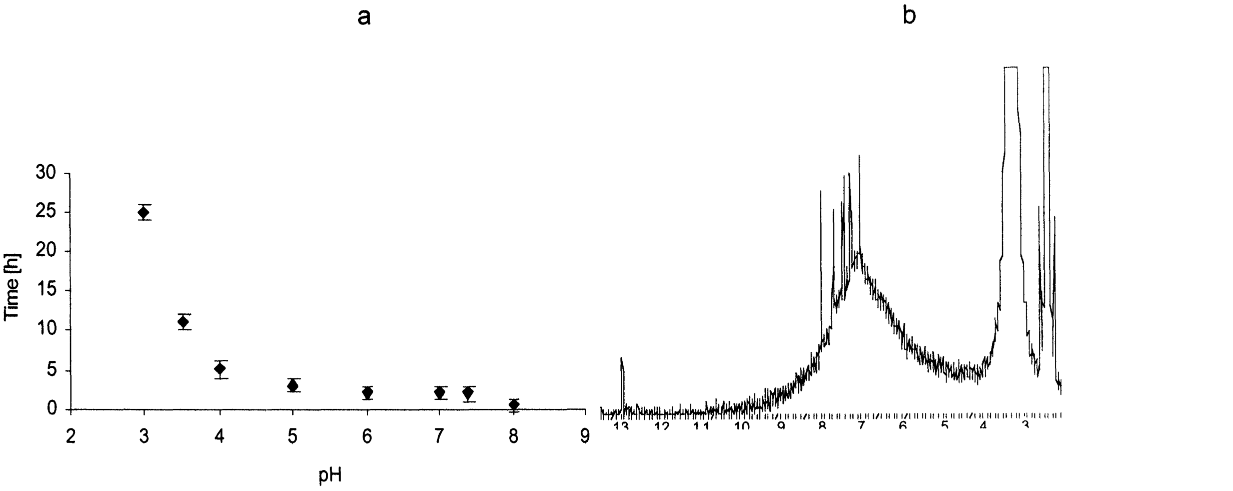

**Figure 6.** a) Time of first appearance of the band at 578 nm in visible spectra of Na *trans*-$[RuCl_4(ind)_2]$ at different pH values of buffered solutions at 37°C; b) $^1H$ NMR in dmso-d6 of the precipitate obtained from a solution of Na *trans*-$[RuCl_4(ind)_2]$ in a buffer consisting of 0.004 M $NaH_2PO_4$, 0.025 M $NaHCO_3$ and 0.1 M NaCl.

### *3.3. Conclusions*

Whereas solvolysis of the complex *trans*-$[RuCl_4(ind)_2]^-$ in organic solvents or aqueous mixtures of solvents like ethanol, acetonitrile and dmso seems to lead for the most part to one main product, hydrolysis in water or buffered aqueous solution is more intricate. Solvolysis in ethanol and acetonitrile / water presumably leads to neutral, monosubstituted ruthenium solvento complexes.

The solvolytic decomposition of the complex *trans*-$[RuCl_4(ind)_2]^-$ is characterized by substitution of one or more of the chloro ligands. An initial formation of the neutral monoaquacomplex $[RuCl_3(H_2O)(ind)_2]$ seems to be necessary for further transformation in all solvent systems as no reaction occurs in absolute dmso and acetonitrile within days. The formation of an aquacomplex is also likely because pH decreases in an unbuffered solution of Na *trans*-$[RuCl_4(ind)_2]$ in water (data not shown). This lowering of pH should be caused by a deprotonation of the formed aquacomplex(es), leading to the corresponding hydroxocomplex(es). The formed hydroxo complexes could then further react to di- or polynuclear hydroxo- or oxo-bridged species, respectively. These reactions should be accelerated at higher pH levels. As we found a strong pH dependence of the hydrolysis, the formation of μ-oxo complexes might be one of the possible reaction pathways. Additionally, a release of the indazole ligands can not be excluded although the Ru(III)-nitrogen bonds in these kind of ruthenium complexes with N-heterocyclic ligands are assumed to be rather inert.
A totally different hydrolysis pathway is followed in the case of the buffer containing $NaHCO_3$. The hydrolysis is much faster and release of indazole could be demonstrated.
This striking influence of $HCO_3^-$ on the hydrolytic decomposition of *trans*-$[RuCl_4(ind)_2]^-$ is important to notice, especially in comparison to the imidazole complex *trans*-$[RuCl_4(im)_2]^-$. Whereas the imidazole complex hydrolyzes faster than the indazole complex in water or phosphate buffer, the situation changes dramatically in physiological buffer containing hydrogen carbonate. A total change in the coordination sphere of the ruthenium complex seems to take place. The formed species react very fast with serum proteins under these conditions [22] (No precipitation occurs if serum proteins are present). Whereas the indazole complex is more stable in water than the imidazole complex, and enough stable with regard to a clinical application, reactions in the blood can proceed much faster compared to the imidazole complex. This might be one of the reasons for the significantly lower toxicity of the indazole complex.

**Acknowledgements**
The assistance of Mrs. Rosa Strobl in preparing the manuscript is gratefully acknowledged. The research is sponsored by the Fonds zur Förderung der wissenschaftlichen Forschung (FWF).

### References

[1] Clarke, M.J. in *Metal Complexes in Cancer Chemotherapy*; Ed. Keppler, B.K.; VCH Weinheim, (1993), 129.
[2] Alessio, E.; Balducci, A.; Lutman, A.; Mestroni, G.; Calligaris, M.; Attia, W.M.; *Inorg. Chim. Acta* (1993), **203**, 205.
[3] Vilaplana, R.; Romero, M.A.; Quiros, M.; Salas, J.M.; Gonzalez-Vilchez, F.; *Metal Based Drugs* (1995), **2**, 211
[4] Mestroni, G.; Alessio, E.; Sava, S.; Pacor, S.; Coluccia M., Boccarelli, A.; *Metal Based Drugs* (1993), **1**, 41.
[5] Keppler, B. K.; Lipponer, K. G.; Stenzel, B.; Kratz, F.; *Metal Complexes in Cancer Chemotherapy*; Ed. Keppler, B.K.; VCH Weinheim, (1993), 187.
[6] Seelig, M.; Berger, M.R. ; Keppler, B.K.; Schmähl, D.; *Metal Ions in Biology and Medicine*; Eds. Collery, Ph.; Poirier, L.A.; Manfait, M.; Etienne, J.C.; John Libbey Eurotext: Paris, (1990), 476.
[7] Galeano, A.; Berger, M.R.; Keppler, B.K.; *Arzneimittelforschung/Drug Research* (1992), **42**(I), 821.
[8] Depenbrock, H.; Schmelcher, S.; Peter, R.; Keppler, B.K.; Weirich, G.; Block, T.; Rastetter, J.; Hanauske, A. R.; *Europ. J. Cancer* (1997), **33**, 2404.
[9] Howe-Grant, M.; Lippard, S.L.; *Metal Ions Biol. Syst.* (1980), **11**, 63.
[10] Hohmann, H.; Hellquist, B.; van Eldik, R.; *Inorg. Chem.* (1992), **31**, 1090.
[11] Hohmann, H.; van Eldik, R.; *Inorg. Chim. Acta* (1990), **174**, 87.
[12] Suvachittanont, S.; van Eldik, R.; *Inorg. Chem.* (1994), **33**, 895.
[13] Holler, E.; Schaller, W.; Keppler, B.K.; *Arzneim.-Forsch. / Drug Res.* (1991), **41**, 1065.
[14] Chatlas, J.; van Eldik, R.; Keppler, B.K.; *Inorg. Chim. Acta* (1995), **233**, 59.
[15] Anderson, C.; Beauchamp, A.L.; *Can. J. Chem.* (1995), **73**, 471.
[16] Ni Dhubhghaill, O.M.; Hagen, W.R.; Keppler, B.K.; Lipponer, K.G.; Sadler, P.J.; *J. Chem. Soc. Dalton Trans.* (1994), 3305.
[17] Lipponer, K.G.; Vogel, E.; Keppler, B.K.; *Metal-Based Drugs* (1996), **3**, 243.
[18] Peti, W.; Pieper, T.; Sommer, M.; Keppler, B.K.; Giester, G.; *Eur. J. Inorg.Chem.* (1999), 1551.
[19] Bertini, I.; Luchinat, C.; *NMR of paramagnetic molecules in biological systems*, Benjamin-Cummings: Menlo Park, CA, (1986).
[20] Satterlee, J.D.; *Concepts Magn. Reson.* (1990), **2**, 69 and (1990), **2**, 119.
[21] Velders, A.H.; Pazderski, L.; Ugozzoli, F.; Biagini-Cingi, M.; Manotti-Lanfredi, A.M.; Haasnoot, J.G.; Reedijk, J.; *Inorganica Chimica Acta* (1998), **273**, 259.
[22] Kratz, F.; Hartmann, M.; Keppler, B.K.; Messori, L.; *J. Biol. Chem.* (1994), **269**, 2581.

**Received: May 22, 2000 - Accepted: August 22, 2000 -**
**Received in revised camera-ready format: August 23, 2000**

